# From SARS-CoV-2 hematogenous spreading to endothelial dysfunction: clinical-histopathological study of cutaneous signs of COVID-19

**DOI:** 10.1186/s13000-021-01075-6

**Published:** 2021-02-25

**Authors:** Angela Patrì, Maria Vargas, Pasquale Buonanno, Maria Carmela Annunziata, Daniela Russo, Stefania Staibano, Giuseppe Servillo, Gabriella Fabbrocini

**Affiliations:** 1 Department of Clinical Medicine and Surgery, Section of Dermatology and Venereology, University of Naples Federico II, Via Pansini 5, Napoli, Italy; 2 Department of Neurosciences, Intensive Care Unit, Reproductive and Odontostomatological Sciences, University of Naples Federico II, Via Pansini 5, Napoli, Italy; 3 Department of Advanced Biomedical Sciences, Pathology Section, University of Naples Federico II, via Pansini 5, Naples, Italy

**Keywords:** Histopathology, Skin, COVID-19, Case report, Endothelial swelling

## Abstract

**Background:**

To date, very few studies on clinical-histopathological correlations of cutaneous disorders associated with COVID-19 have been conducted.

**Case presentation:**

The Case 1 was a 90-year-old man, who tested positive for SARS-CoV-2 from a nasopharyngeal swab. Two days later, he was hospitalized and after eleven days transferred to Intensive Care Unit. A chest CT showed bilateral ground-glass opacities. Just that day, an erythematous maculo-papular rash appeared on trunk, shoulders and neck, becoming purpuric after few days. Histological evaluations revealed a chronic superficial dermatitis with purpuric aspects. The superficial and papillary dermis appeared edematous, with a perivascular lympho-granulocytic infiltrate and erythrocytic extravasation. At intraepithelial level, spongiosis and a granulocyte infiltrate were detected. Arterioles, capillaries and post-capillary venules showed endothelial swelling and appeared ectatic. The patient was treated with hydroxychloroquine, azithromycin, lopinavir-ritonavir and tocilizumab. Regrettably, due to severe lung impairment, he died.

The Case 2 was a 85-year-old man, admitted to Intensive Care Unit, where he was intubated. He had tested positive for SARS-CoV-2 from a nasopharyngeal swab two days before. A chest RX showed bilateral atypical pneumonia. After seven days, a cutaneous reddening involving trunk, upper limbs, neck and face developed, configuring a sub-erythroderma. Histological evaluations displayed edema in the papillary and superficial reticular dermis, and a perivascular lymphocytic infiltrate in the superficial dermis. The patient was treated with hydroxychloroquine, azithromycin, lopinavir-ritonavir and tocilizumab. Sub-erythroderma as well as respiratory symptoms gradually improved until healing.

**Conclusions:**

The endothelial swelling detected in the Case 1 could be a morphological expression of SARS-CoV-2-induced endothelial dysfunction. We hypothesize that cutaneous damage could be initiated by endothelial dysfunction, caused by SARS-CoV-2 infection of endothelial cells or induced by immune system activation. The disruption of endothelial integrity could enhance microvascular permeability, extravasation of inflammatory cells and cytokines, with cutaneous injury. The Case 2 developed a sub-erythroderma associated with COVID-19, and a non-specific chronic dermatitis was detected at histological level. We speculate that a purpuric rash could represent the cutaneous sign of a more severe coagulopathy, as highlighted histologically by vascular abnormalities, while a sub-erythroderma could be expression of viral hematogenous spreading, inducing a non-specific chronic dermatitis.

## Background

An increasing number of reports on skin involvement in patients affected with Coronavirus Disease 2019 (COVID-19) is currently available. However, very few studies on clinical-histopathological correlations of skin disorders associated with COVID-19 have been conducted [1–5]. Different clinical features of cutaneous involvement in patients affected with SARS-CoV-2 infection seem to reveal a full spectrum of viral interaction with the skin [1]. Herein, we report two COVID-19 patients developing a purpuric maculo-papular rash and a sub-erythroderma, respectively.

## Case presentation

**Fig. 1:**
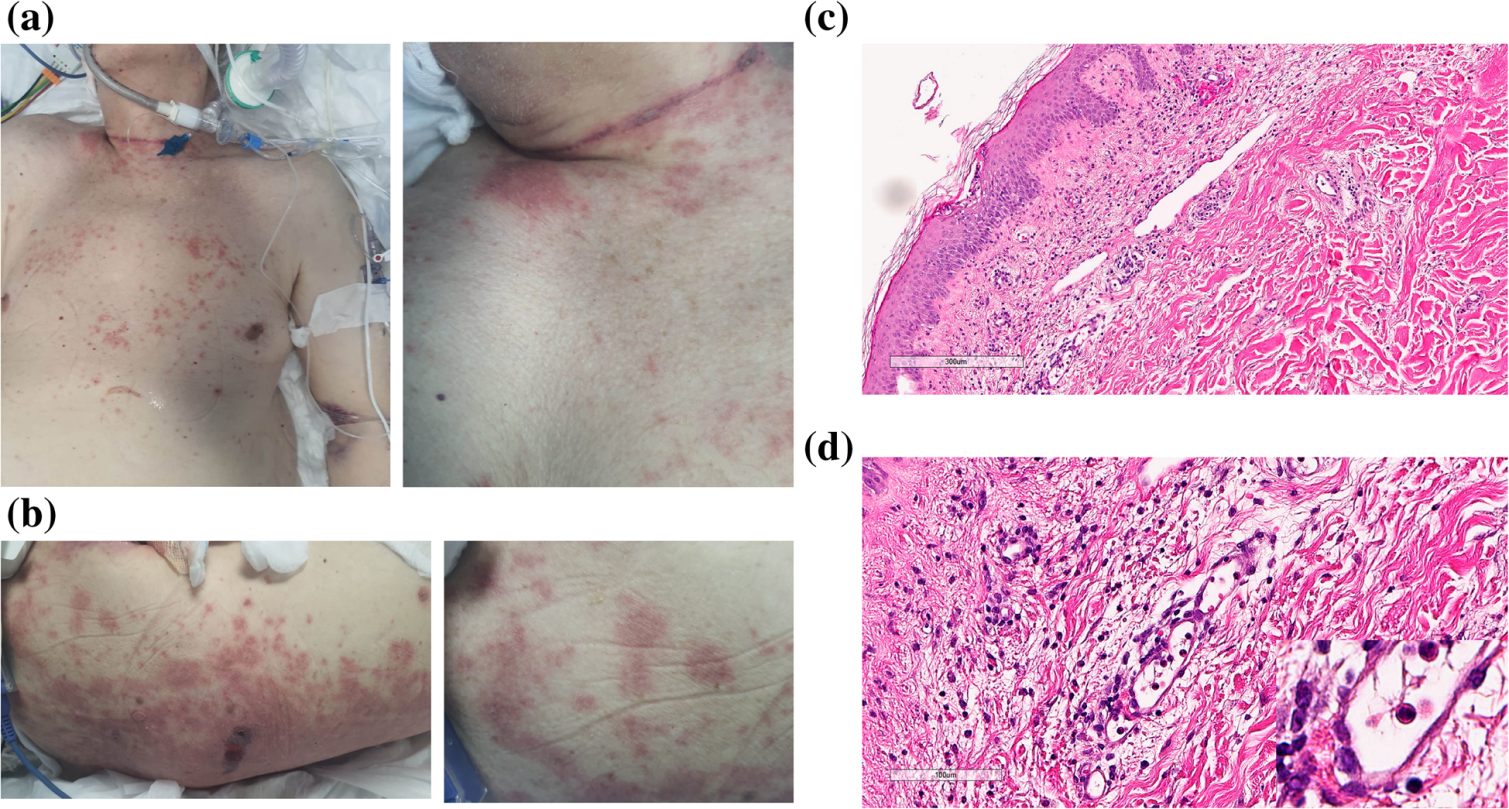
**a** Purpuric maculo-papular rash involving trunk, neck and shoulders; **b** Back lesions with limited epidermal detachment; **c**-**d** The papillary and superficial dermis were edematous and populated mainly by perivascular lympho-granulocytic inflammatory infiltrate, with associated granulocyte intraepithelial exocytosis, and irregular spongiosis (blue arrow). Superficial dermal micro-areas of erythrocytic extravasation (yellow arrow) and endothelial “swelling” (green arrow in fig. **c** and fig.**d**; at higher magnification in inset on the right) were observed (C:H&E, 8x; D,:H&E, 20x, inset 40x). Legend: H&E = hematoxylin and eosin stain

The Case 1 was a 90-year-old man, affected with hypertension and senile dementia, who tested positive for SARS-CoV-2 by reverse transcriptase–polymerase chain reaction (RT-PCR) from a nasopharyngeal swab on April 2th, 2020, after three days of fever and cough. On April 4th he was hospitalized and after eleven days transferred to Intensive Care Unit (ICU) due to the severe dyspnea, requiring intubation. A chest CT showed bilateral ground-glass opacities in upper and lower lobes. Just that day, an erythematous maculo-papular rash appeared on trunk, shoulders and neck, becoming purpuric after few days (Fig. 1a,b). On April 23th, five biopsy specimens from the back and upper limbs were obtained. Histological evaluations revealed a chronic superficial dermatitis with purpuric aspects. The superficial and papillary dermis appeared edematous, with a perivascular lympho-granulocytic infiltrate and erythrocytic extravasation. At intraepithelial level, spongiosis and a granulocyte infiltrate were detected. Arterioles, capillaries and post-capillary venules showed endothelial swelling and appeared ectatic (Fig. 1c-d). The patient was treated with hydroxychloroquine, azithromycin, lopinavir-ritonavir and tocilizumab. Regrettably, due to severe lung impairment, he died on April 25th.

**Fig. 2:**
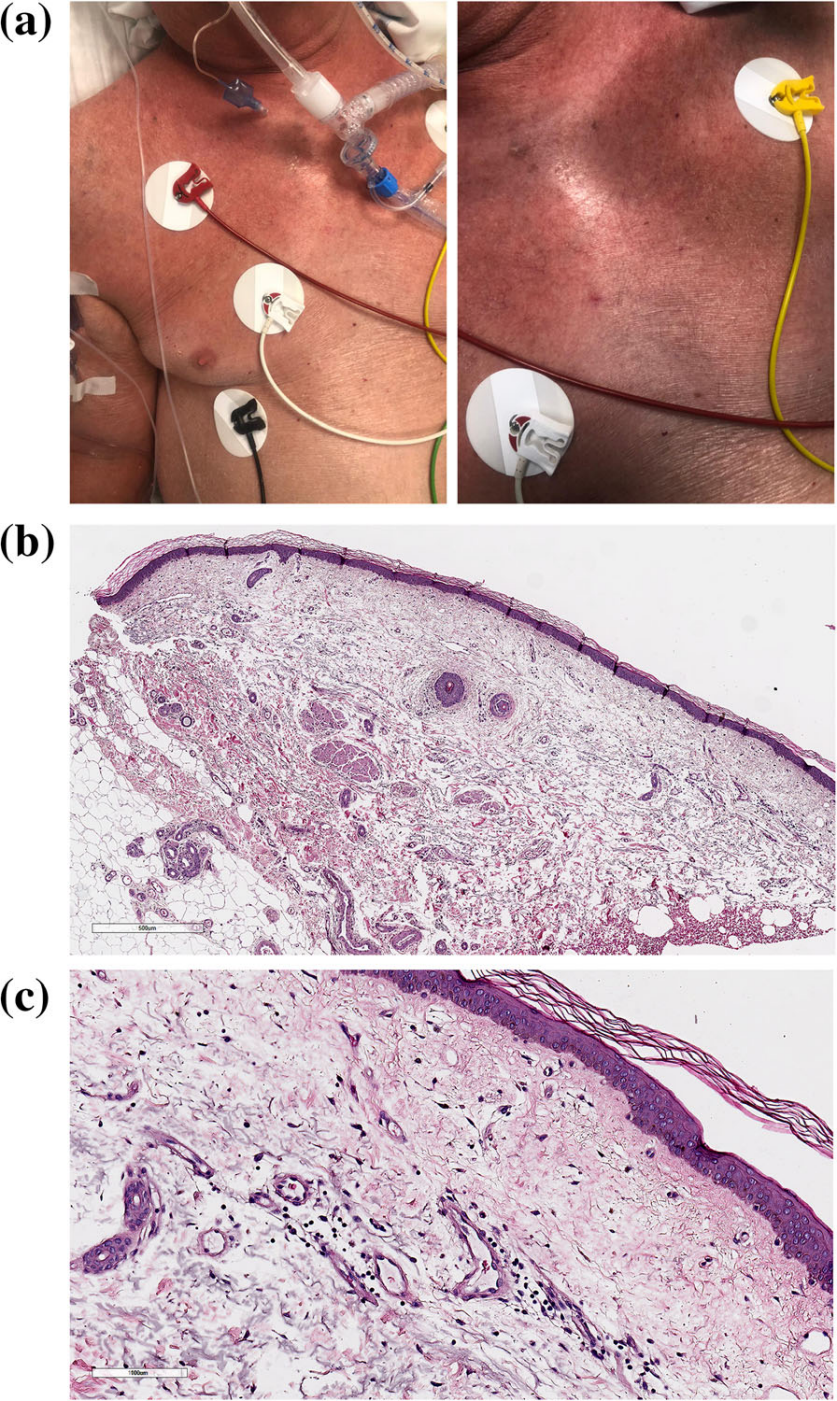
**a** Sub-erythroderma with mild exfoliation, affecting trunk, upper limbs, neck and face; **b**, **c** Edema and moderate chronic lymphocytic inflammation, mainly perivascular, were present in the papillary and superficial reticular dermis (A:H&E, 5x; B:H&E, 20x)

The Case 2 was a severely dyspneic 85-year-old man, with a history of hypertension, cerebral vasculopathy, prostate cancer, admitted to ICU on April 19th, where he was intubated. He had tested positive for SARS-CoV-2 by RT-PCR from a nasopharyngeal swab on April 17th, after five days of fever, cough and sore throat. A chest RX showed bilateral atypical pneumonia. On April 24th, a cutaneous reddening involving trunk, upper limbs, neck and face developed, configuring progressively a sub-erythroderma, with mild exfoliation (Fig. 2a). After three days, four biopsy specimens were obtained. Histological evaluations displayed edema in the papillary and superficial reticular dermis, and a perivascular lymphocytic infiltrate in the superficial dermis (Fig. 2b,c). The patient was treated with hydroxychloroquine, azithromycin, lopinavir-ritonavir and tocilizumab. Sub-erythroderma as well as respiratory symptoms gradually improved until healing, with the hospital discharge occurring on May 5th.

## Discussion and conclusions

Our first patient showed similarities with a case of purpuric rash reported by Gianotti R et al [1].Common histological features were lymphocytic infiltration around the swollen blood vessels with extravasated erythrocytes and spongiosis [1].Dilated vessels were also found by Zengarini C et al. [3] in an erythematous rash associated with COVID-19 [3].In addition to ectatic vessels, our Case 1 showed endothelial swelling. Such feature could be a morphological expression of SARS-CoV-2-induced endothelial dysfunction. Indeed, SARS-CoV-2 can infect cells by angiotensin-converting enzyme 2 receptor, largely expressed in endothelial cells [6, 7].An endotheliitis has been hypothesized as the origin of compromised microcirculation affecting lungs and kidneys in COVID-19 [7].Endothelial cells’ infection could trigger activation of coagulation and diffuse microthrombosis [7].Thus, we hypothesize that cutaneous damage could be initiated by endothelial dysfunction, caused by SARS-CoV-2 infection of endothelial cells or induced by immune system activation. The disruption of endothelial integrity could enhance microvascular permeability, extravasation of inflammatory cells and cytokines, with cutaneous injury.

To the best of our knowledge, our Case 2 is the first report displaying histological features of a sub-erythroderma associated with COVID-19. A non-specific chronic dermatitis was detected, as often found in erythroderma from other causes. The reason why cutaneous manifestations can be so different among patients is unknown. However, it is clear that COVID-19 can lead to coagulopathy. During hospitalization, both our patients had high D-dimer levels, which were on average 3-fold higher in Case 1 in comparison with Case 2. We speculate that a purpuric rash could represent the cutaneous sign of a more severe coagulopathy, as highlighted histologically by vascular abnormalities, while a sub-erythroderma could be expression of viral hematogenous spreading, inducing a non-specific chronic dermatitis, also in absence of histopathological microangiopathy signs.

## Data Availability

Data sharing is not applicable to this article as no datasets were generated or analysed during the current study.

## Abbreviations

COVID-19: Coronavirus Disease 2019
RT-PCR: Reverse transcriptase–polymerase chain reaction
ICU: Intensive Care Unit
